# Cognitive Training for Schizophrenia in Developing Countries: A Pilot Trial in Brazil

**DOI:** 10.1155/2013/321725

**Published:** 2013-10-30

**Authors:** Livia M. M. Pontes, Camila B. Martins, Isabel C. Napolitano, Juliana R. Fonseca, Graça M. R. Oliveira, Sandra M. K. Iso, Anny K. P. M. Menezes, Adriana D. B. Vizzotto, Elaine S. di Sarno, Hélio Elkis

**Affiliations:** ^1^Department and Institute of Psychiatry, University of São Paulo Medical School, Rua Ovídio Pires de Campos, 785, 05403-010 São Paulo, SP, Brazil; ^2^Institute of Mathematics and Statistics, University of São Paulo, Rua do Matão, 1010, 05508-090 São Paulo, SP, Brazil; ^3^Schizophrenia Programme, Institute of Psychiatry, University of São Paulo General Hospital, Rua Ovídio Pires de Campos, 785, 05403-010 São Paulo, SP, Brazil; ^4^Integrated Mental Health Care Centre of Santa Casa de Misericórdia of São Paulo Hospital, Rua Major Maragliano, 241, 04017-030 São Paulo, SP, Brazil

## Abstract

Cognitive deficits in schizophrenia can massively impact functionality and quality of life, furthering the importance of cognitive training. Despite the development of the field in Europe and in the United States, no programmes have been developed and tested in developing countries. Different cultural backgrounds, budget restrictions, and other difficulties may render treatment packages created in high income countries difficult for adoption by developing nations. We performed a pilot double-blind, randomized, controlled trial in order to investigate the efficacy and feasibility of an attention and memory training programme specially created in a developing nation. The intervention used simple, widely available materials, required minimal infrastructure, and was conducted in groups. The sample included seventeen stable Brazilians with schizophrenia. Sessions were conducted weekly during five months. The cognitive training group showed significant improvements in inhibitory control and set-shifting over time. Both groups showed improvements in symptoms, processing speed, selective attention, executive function, and long-term visual memory. Improvements were found in the control group in long-term verbal memory and concentration. Our findings reinforce the idea that cognitive training in schizophrenia can be constructed using simple resources and infrastructure, facilitating its adoption by developing countries, and it may improve cognition.

## 1. Introduction

Impaired cognitive functioning in schizophrenia was early recognized [1, 2] and described as one of the core symptoms [3]. Deficits may be present since the first manifestations of the disease or may appear before the onset of symptoms [4]. Up to 80% of all people with schizophrenia may present significant deficits [5].

Main deficits involve attention, memory, and executive functions, but other domains can also be affected, such as visuospatial ability, language, learning, and motor coordination [3, 6, 7]. Cognitive deficits have been related to impairment in functional abilities [8–10], compromising the ability to lead an independent life, to benefit from psychosocial treatments, to create and maintain social relationships, to find and keep a job, and to maintain academic development [6, 11–13]. Improvements in cognitive functions can increase benefits from other psychosocial rehabilitation programmes [14]; therefore, treatment of schizophrenia could also include cognitive training.

A large number of outpatient consultations and re-hospitalization rates [15], difficulty in maintaining a job [6], and claims for sickness/disability benefits at an early age [16] are some factors that place schizophrenia amongst the most costly illnesses in the world [17]. In Brazil, cost estimates for the public health system in the most economically developed State (São Paulo) indicate that schizophrenia costs correspond to 2.2% of its total expenditure on health [18].

Systematic reviews and meta-analysis [19, 20] have shown that cognitive training is effective for the improvement of cognition in schizophrenia, thus enabling people to return to work or school.

Despite the fact that guidelines published by high income countries, The Schizophrenia Patients Outcome Research Team (PORT) [21] in the USA and the National Institute for Health and Clinical Excellence (NICE) in the UK [22], did not recommend cognitive remediation as an effective treatment, the largest and most updated meta-analysis [23] indicated durable and positive effects on cognition and psychosocial functioning. Connections between results and aspects of the treatment (type of approach used and the use of computerized resources) were not found. Clinically stable persons seem to benefit more from interventions, and improvement in functioning is enhanced when cognitive training is used together with psychiatric interventions. Methodological rigor was not found to be related to treatment results as the most rigorous studies found only small to moderate effects. The type of control condition (active versus passive) also does not interfere with cognitive outcomes [23].

Even in low- and middle-income countries (LAMICs), a recent report [24] indicated that cognitive remediation is more cost-effective than usual care in improving working memory and executive functioning at no additional cost.

Numerous treatment programmes and studies have been created and conducted in the United States and Europe [23, 25]. To the best of our knowledge, no programmes have been created and tested in developing countries. It is estimated that 25 [26] to 41.7 million people [27] are affected by schizophrenia in LAMIC; hence, the majority of persons affected live in such countries [28].

Developing countries suffer from a lack of psychosocial interventions for treating many mental health conditions and a lack of qualified mental health professionals [26, 29–31]. Suggestions to tackle some of these difficulties include training nonspecialist health professionals to deliver psychosocial interventions [29, 30], employment of inexpensive technology, and programmes conducted in groups, which not only are more cost effective but can help address social skills' difficulties in schizophrenia [32].

These aspects stress the need to create treatment programmes adapted to the challenges faced by LAMIC. Additionally, changes in clinical practice in a developing country are more likely to be adopted by policy makers when the research is conducted in that specific country than when conducted in a high income country [33].

This study aims to investigate the efficacy and feasibility of a noncomputerized attention and memory training programme for people with schizophrenia, created in a developing country. The programme employed simple and easily accessible materials, was constructed to be delivered in groups, requires minimal infrastructure, and can be facilitated by different trained mental health professionals. Our intent is also to raise awareness of the necessity of more research on the adoption of cognitive remediation programmes for schizophrenia in developing countries.

## 2. Methods

### 2.1. Study Locale and IRB Approvals

This study was performed at the Schizophrenia Programme at the Institute of Psychiatry of the University of São Paulo Medical School (FMUSP) and at the Integrated Mental Health Care Centre of the Santa Casa de Misericórdia of São Paulo Hospital. Institution Review Boards of both University hospitals approved the protocol (University of São Paulo General Hospital—Protocol no. 1122/07; Santa Casa de Misericórdia of São Paulo Hospital—Protocol number 162/09).

### 2.2. Selection of Participants

Eligible participants were assessed by experienced psychiatrists and had to fulfill the diagnostic criteria for schizophrenia according to the DSM-IV-R, be between 18 and 50 years of age, be under treatment with atypical antipsychotic medication, be clinically stable (i.e., a score of 60 or less on the positive and negative syndrome scale for schizophrenia—PANSS) [34], be fluent in written Portuguese, and agree to sign the informed consent form. Exclusion criteria were a history or evidence of neurological conditions (e.g., epilepsy or brain trauma); an estimated I.Q. (as measured by Vocabulary and Block Design subtests of Wechsler Adult Intelligence Scale Revised—WAIS-R) [35] under 70 (i.e., below the borderline range); current dependency on alcohol or psychoactive substances, except tobacco; and participation in any cognitive training programme in the previous six months.

**Figure 1:**
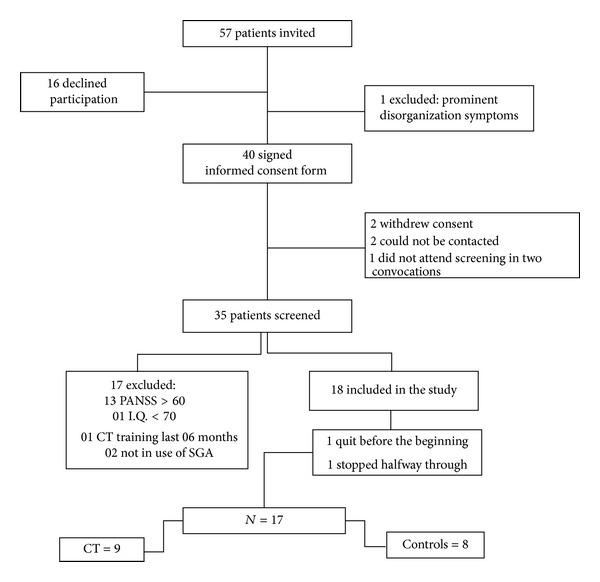
Consort diagram. CT indicates cognitive training; SGA indicates second generation antipsychotic.

Outpatients of both centres were asked by their psychiatrists to voluntarily take part in the research. Fifty-seven outpatients were initially invited. Forty volunteers gave their written consent to take part in the trial. One person was excluded due to the presence of prominent disorganization symptoms. Thirty-five people were screened, and eighteen were included. One participant got a job and had to stop halfway through the study, but her data were included in the analysis (intention-to-treat approach). The final sample comprised seventeen participants. These aspects are illustrated in the CONSORT Diagram, in Figure 1, as well as reasons for not meeting eligibility criteria. 

As we aimed to pilot test a treatment programme, there was no sample size calculation.

### 2.3. Assessments

Two of the authors (LMMP and ADBV) interviewed volunteers for eligibility criteria and demographic data on the first visit. Subjects who met inclusion criteria underwent symptoms and I.Q. evaluations. If the determined scores were achieved, volunteers were sent for cognitive assessment, which was done both at baseline and after intervention and included Trail Making Test [36], Stroop Color Naming Test [36], Modified Wisconsin Card Sorting Test [37, 38], and Logical Memory and Visual Reproduction subtests from the Wechsler Memory Scale III [39].

PANSS evaluations were carried out either by masked psychiatrists or psychologists, who were trained and had a minimum intraclass correlation coefficient of *r* = 0.80. All the other instruments were applied by masked neuropsychologists who had specialists' degrees, trained by the Psychology Department of the Institute of Psychiatry at the University of São Paulo Medical School.

Block randomization was generated via the randomization.com website [40] by a professional who was not involved in the study. This professional created cards designed to assign individuals to one of two conditions: group 1, the experimental group, which received the cognitive training (CT) or group 2, the control condition group, which received reading training.

Although it has been reported that masking allocation of participants has no effect on cognition or functioning in cognitive training in schizophrenia [23], in order to guarantee the double-blind design, volunteers were told that they would be drawn by lot to take part in one of two kinds of cognitive training. If one of the treatments proved superior to the other, the person could take part in this treatment after the study was over.

Medication dosage was adjusted at the discretion of each participant's psychiatrist and was not controlled throughout the study.

### 2.4. Cognitive Attention and Memory Training

Training was conducted by the first author in both treatment centres. It comprised 20 group sessions, in weekly frequency, lasting between 40 and 60 minutes, over 5 months. Although our programme was shorter than the average reported in the literature (32.2 hours divided over 16.7 weeks) [23], we wanted to test the efficacy of a shorter programme (20 hours, over 20 weeks) due to important hurdles of longer and more intensive treatment programmes in a developing country. Requiring patients to come to the hospital more than once a week is problematic, as patients (a) have limited budgets for transportation, (b) generally live far from treatment centres and (c) there is a limited workforce and a great number of patients, which necessitates mental health professionals being involved in different duties, making it difficult for them to dedicate more time only to one treatment programme. 

This programme was created according to the cognitive neuropsychology theory (understanding of the normal performance of the cognitive function to be trained), and cognitive retraining approaches (stimulation of cognitive functions through drill and practice) [41]. It included restorative activities (repetition and practice) and compensatory strategies. Considering that motivation is an important aspect of schizophrenia and can interfere with treatment compliance, activities were developed to be pleasurable and motivating. 

Sessions were constructed hierarchically by level of difficulty and were planned to resemble daily activities. Infrastructure demands were minimal: one room large enough to fit 10 people, a table and chairs to accommodate participants and therapist, paper and pencil for participants, and prepared worksheets. Easily accessible materials were used, for example, newspaper headlines, books, magazine pictures, playing cards, music, short stories, supermarket lists, pictures of clothes, objects, and fruits. Activities and worksheets were prepared by two authors (LMMP, HE) and organized in a manual [42].

The first 10 sessions focused on the training of attention, as this is a basic cognitive function that supports many other functions, including memory [43]. The last 10 sessions concentrated on memory training. Each activity was used in two sessions in order to propitiate a better learning and cognitive efficiency. 

 Important aspects of empirically tested programmes were incorporated: metacognition, contextualisation, verbalisation, and positive reinforcement [44–46].

The promotion of metacognition through questions about the use of cognition promotes analogical reasoning (i.e., the ability to recognize similarities between tasks), self-regulation, and a more active participation, facilitating learning and generalisation [44, 45]. Contextualisation of activities seems to promote motivation, as the practical usefulness of the activity and its connection to real life situations is made clear [46]. At the beginning of each session, it was explained to participants which cognitive function was going to be practiced and where such function was generally used in daily activities (contextualisation). They were then stimulated (through questions) to think about how they used the function in their lives, difficulties encountered, and the importance of good performance of such function (metacognition). From the second session on, they were also questioned whether they used the previously taught strategies. Barriers to the use (lack of opportunity, forgetfulness, misunderstanding of the strategy, seeing the strategy as too difficult, etc.) were discussed (metacognition).

Verbalisation was taught and stimulated throughout the training as it facilitates information processing and memorisation. Positive reinforcement was also used to acknowledge participants' involvement and effort in the training, as it has shown to be effective in this population [44].

As memory impairment in schizophrenia seems to be related to difficulties in organising the material to be remembered [6], for the memory training two main organisation procedures were used: categorisation and expanded rehearsal [47, 48].

No frequency criterion was used, but absences were checked by telephone, and the person was encouraged to attend the next session.

Detailed description of each activity can be provided by the first author upon request. 

### 2.5. Control Group

The same number, frequency, and duration of sessions were employed in the control group, and training was also conducted by the first author. Training comprised reading activities (magazines or newspapers articles). In order to resemble cognitive training, allow participant blinding, and match groups for professional and participant interaction and motivational aspects, articles were also hierarchically arranged by difficulty level (beginning with shorter ones and moving on to longer ones). A strategy was taught: first the title, subtitles, pictures captions, and charts/tables were read aloud by the therapist, and participants were encouraged to express their views on what was going to be covered by the article. Then, the whole article was read, and participants were requested to express their understanding of the theme and give their opinion on the subject. Texts were presented in the same order to all control groups. Themes involving schizophrenia-related issues were avoided so that this intervention would not resemble psychotherapy.

Absences were also checked by telephone, and the person encouraged to attend the next session. 

### 2.6. Statistical Analysis

Data were analysed according to an intention-to-treat approach and the last observation carried forward of the participant who stopped training halfway through was included in this analysis. Only nonparametric tests were used, and this decision was made on two main pillars: (1) the sample size was very small, and nonparametric tests are less sensitive to outliers; (2) non-parametric tests are suitable for analyses of continuous, ordinal variables [49].

For baseline comparisons between groups, the Mann-Whitney Test was used for the continuous variables, and the Chi-squared test was used for the categorical variables. For cognitive performance comparisons between the experimental and control groups at different moments (baseline and after intervention), a nonparametric analysis of repeated measures [49–52] was used. This method tests the hypotheses of the existence of an effect either between groups (CT and control) or within groups (repetitions-changes over time), as well as the interaction between these factors (groups and changes over time). Detailed descriptions of this method can be obtained elsewhere [49–51, 53].

In order to verify the effect-sizes, Cohens'd [54, 55] were also calculated considering the difference between after intervention and baseline scores.

## 3. Results

Groups were matched for demographic and clinical characteristics at baseline, except for Visual Reproduction II, in which the experimental group had a higher score than the control group. Treatment attendance indicated that although the control group had a higher mean of attendance than the experimental group such difference was not significant (Table 1).

After five months of cognitive training, significant differences were found within groups both on the positive (*P* = 0.012) and the general psychopathology (*P* = 0.044) subscales of the PANSS, indicating that over time, the symptoms of both groups had improved (Table 2).

Regarding attention measures (Table 2), both groups improved significantly in processing speed (Trail Making A, *P* = 0.003), but the interaction effect (*P* = 0.019) indicates that the control group had a larger improvement than the CT group, with a large effect-size (*d* = 0.821). In Trail Making B, significant effects were found within groups in set-shifting (Trail Making B, *P* = 0.045), indicating an improvement in the CT group over time and a decrease in the control group. In the Stroop Test, both groups presented a significant improvement in selective attention over time (Card II, *P* = 0.001), but the absence of an interaction effect does not allow for a comparison between the improvements of the groups. A significant interaction effect favouring the CT group was found in the Stroop Test card III (*P* = 0.036), indicating an improvement in inhibitory control, with a large effect-size (*d* = 1.549). Regarding executive functioning, both groups showed an improvement in perseverative errors on the MWCST in their scores over time (*P* = 0.046), but no interaction effect was found, precluding a comparison of improvement between the two groups. A significant interaction effect favouring the control group was found in concentration (MWCST failure to maintain set, *P* = 0.010), with a large effect-size (*d* = 1.086).

Memory assessments (Table 2) indicated no significant differences in short-term verbal and visual memories (Logical Memory I and Visual Reproduction I). A significant interaction effect and a large effect-size favouring the control group were found in long-term verbal memory (Logical Memory II, *P* = 0.003; *d* = 1.618). This indicates that while the CT group's performance deteriorated, the control group's improved. A significant improvement was found within groups in long-term visual memory (Visual Reproduction II, *P* = 0.072), but no differential effects were found in interaction, indicating that although both groups improved over time none of the groups improved more than the other.

## 4. Discussion

Our noncomputerized cognitive training programme seems to significantly improve performance in a cognitive measure of attention, inhibitory control, as measured by the Stroop Color Naming Test [36, 56], with a large effect-size. However, these results need further investigation in order to be corroborated. A review [57] defines positive results as a significant improvement in at least one outcome measure in relation to the control group, reinforcing the impression that our results are positive, albeit still very modest. As described in the literature, attention and information processing deficits are associated with functional impairment, especially in social functioning [6, 58]. Inhibitory control is much applied to social situations; it prevents impulsive reactions and enables distracting stimuli to be inhibited so that attention can be better focused. Therefore, an improvement in inhibitory control can represent moderate to large functional gains, which could not be verified in this study because a functional measure of attention was not available in Portuguese.

Set-shifting was also improved in the CT group, whereas it deteriorated in the control group. This can be understood by the fact that while this function was trained for four sessions in the CT group, the reading of texts in the control group did not require attention shifting; on the contrary, attention focus was maintained on the same theme throughout the session.

Other findings related to attention, although positive, did not show significant differences between groups (selective attention, executive function) or indicated a greater improvement of the control group (processing speed and concentration). They may indicate that the reading intervention was beneficial to these cognitive functions, although this cannot be generalized due to the small sample size. The reading intervention seems to have taught participants to process initial information, gradually directing their attention to the theme and organizing the information to be learned (demanding on executive function). The gradation of texts demanded the development of processing speed and selective attention, because more information had to be processed, and attention had to be focused longer in each session.

Regarding memory effects, information storage seems to be preserved in schizophrenia, as shown in recognition tasks [59], but retrieval of both immediate and delayed information has been described as the most impaired memory function and also considered a trait of this disorder [60]. A significant result was found on long-term verbal memory in favour of the control group. These results can be understood when one considers that in the control group both short-term and long-term verbal memories were evoked by the reading of texts and subsequent discussion between participants. Furthermore, participants sometimes commented on the theme read in the previous sessions, and family members reported that they would also make observations about the text later at home, indicating that long-term verbal memory was being exercised. The decrease in long-term verbal memory in the CT group can be understood in the light of utilization deficiency [61, 62], which considers that the use of a memory strategy may not lead to benefits in recall, and it can even lower information retrieval. This can occur because, although the strategy might be used correctly, it might be employed in a simpler or less effective manner. Some factors seem to account for this: the availability of mental resources (whenever a new strategy is taught, most mental effort is concentrated on the execution of the strategy, leaving few resources to be allocated to the recall of information); previous knowledge (it is easier to recall familiar rather than unfamiliar information); co-occurrence of one or more strategies; differences in metamemory; difficulty in inhibiting the use of an ineffective strategy learned previously; and other factors, such as motivation and self-efficacy. However, utilization deficiency is considered in a continuum in the development of strategies as the most sophisticated one, frequently preceding significant benefits with the use of the strategy. This might be an indication that further training of verbal memory could have resulted in larger improvements.

Treatment attendance showed that both interventions had similar acceptability rates among participants, indicating that the cognitive exercises proposed were as engaging and motivating as structured reading activities. Nevertheless, the present effects were observed in individuals who on average attended about 14 sessions (out of 20) of training and may be different for individuals who are more/less adherent to the intervention. Although no significant differences were found in attendance, it is possible that participating in more sessions contributed to the positive findings of the control group.

Neuropsychological tests might not be sensitive enough to capture smaller improvements in cognition, so the uses of functional measures are encouraged [10]. At the time of data collection, there was no functional measure available in Portuguese. Recently, an instrument has been translated and adapted for Portuguese [63], Direct Assessment of Functional Status (DAFS-BR).

Receiving professional attention, feeling cared for, and interacting socially may contribute to the lowering of anxiety, which leads to an improvement in cognition and symptoms [11]. These factors may have contributed to the improvement in symptoms and cognition found in both groups.

Although medication effects cannot be completely ruled out on the improvement of symptoms, participants were stable from the onset of treatment, so any medication effect might have reached its peak before the commencement of the trial.

This study contains significant limitations which means that it is not yet possible to recommend or justify its adoption. The first one is that although this is a controlled trial, the final sample is quite small, with limited statistical power to arrive at more robust conclusions and generalisations.

Secondly, although data points to the superiority of second generation antipsychotics (SGAs) in a series of cognitive domains [64], some volunteers were also using other types of drugs including anticholinergic medication, which is known to interfere with cognitive functioning [6]. However, any interference may have affected both groups equally.

Thirdly, a learning effect with neuropsychological instruments cannot be excluded, but such an effect is likely to be modest after five months and to affect both groups equally.

In fourth place, it seems that the control condition led to some degree of cognitive training, as it shared elements with the experimental group; it demanded the use of several cognitive functions, and it comprised strategies that seemed to train cognition somewhat (strategy use, prompts to aid attention, and comprehension exercises). The inclusion of an active control condition was chosen because it facilitated participant masking and allowed groups to be matched for important cognitive training characteristics: gradation of difficulty level and motivation. It has been proposed that active control conditions are important in order to identify effects that are specific to the treatment programme [21, 23]. Nevertheless, reading activities have been used as part of a cognitive training programme for schizophrenia [65], reinforcing the notion that this activity might favour cognitive performance in this population. Additionally, negative results for cognition have been found in a recent study where an active control condition was used [21], highlighting the need for replication of our research with a passive control condition. The inclusion of both active and passive control conditions is important as it allows for the identification of important treatment elements and whether the treatment programme is effective and worth investing in [23].

In fifth place, as there was no follow-up period, the durability of the findings cannot be assessed.

Finally, even though we adopted the standard significance level of 5% (*α* = 0.05) the possibility of a Type I error is present.

## 5. Conclusions

To our knowledge, this was the first research aimed to pilot test a new cognitive training programme for schizophrenia specifically designed for a developing country. However, it is possible that studies were missed due to the lack of indexation of LAMIC journals in major databases [66] or to the hurdles in publishing research on mental health conducted in LAMIC [32].

Our programme can address some of the recommendations made for treatment interventions for schizophrenia in LAMIC: (a) it is affordable, feasible, and acceptable [26]; (b) it can be facilitated by nonspecialist health professionals [29, 30]; (c) it uses inexpensive resources and can be conducted in groups [32]; and (d) it has an underlying rationale with a structured environment, essential to the rehabilitation of severe mental disorders [29].

The present findings, despite the small sample size, seem promising, due to the methodological rigor of our study.

Recommendations for future studies include a larger sample, preferably with sample size calculation, the use of a passive control condition, and the inclusion of a functional assessment measure.

## Figures and Tables

**Table 1:** Demographic and clinical characteristics at baseline and treatment attendance.

	Experimental *n* = 9	Control *n* = 8	*P*
Institution (% SPU)	66.7	75	1.000°
Gender (male %)	89	75	0.576°
Age (mean ± SE)	37.1 ± 2.7	39.3 ± 2.3	0.735*
Duration of illness (mean ± SE)	15.1 ± 2.6	15.2 ± 2.3	0.885*
Number of previous hospitalizations (mean ± SE)	3.1 ± 1.1	1.1 ± 0.4	0.124*
Years of education (mean ± SE)	10 ± 4.8	10.7 ± 4.5	1.000*
I.Q. (mean ± SE)	93.3 ± 4.3	94.2 ± 4.8	0.961*
PANSS negative subscale (mean ± SE)	11.0 ± 1.3	11.7 ± 1.1	0.697*
PANSS positive subscale (mean ± SE)	12 ± 1.1	11.2 ± 1.1	0.699*
PANSS general psychopathology subscale (mean ± SE)	24.4 ± 1.2	25.7 ± 1.5	0.409*
PANSS total (mean ± SE)	47.4 ± 2.8	48.7 ± 2.8	0.735*
Trail making test A (mean ± SE)	57.2 ± 13.7	59.0 ± 12.0	0.630*
Trail making test B (mean ± SE)	155.8 ± 29.8	121.1 ± 21.2	0.564*
Stroop test card I (mean ± SE)	18.5 ± 1.5	19.2 ± 2.9	0.772*
Stroop test card II (mean ± SE)	28.1 ± 5.1	22.2 ± 3.7	0.359*
Stroop test card III (mean ± SE)	29.8 ± 2.9	27.6 ± 3.5	0.441*
MWCST total categories (mean ± SE)	4.3 ± 0.6	4.1 ± 0.6	0.883*
MWCST perseverative errors (mean ± SE)	8.1 ± 3.2	8 ± 3.5	1.000*
MWCST failure to maintain set (mean ± SE)	0.3 ± 0.2	0.6 ± 0.3	0.406*
Logic memory I (mean ± SE)	14.4 ± 2.7	13.7 ± 3.8	0.847*
Logic memory II (mean ± SE)	10.6 ± 2.2	8.9 ± 3.9	0.286*
Visual reproduction I (mean ± SE)	34 ± 2.9	29.7 ± 3.4	0.225*
Visual reproduction II (mean ± SE)	28.7 ± 3.3	13.5 ± 3.2	0.016*
Treatment attendance (mean ± SE)	14.6 ± 1.7	17.1 ± 0.7	0.481*

SE: standard error, °Fisher's exact test, and *Mann-Whitney test for independent samples.

SPU: Schizophrenia Project Unit at the Institute of Psychiatry of University of São Paulo (USP) Medical School.

PANSS: positive and negative syndrome scale for schizophrenia, MWCST: modified Wisconsin card sorting test.

**Table 2:** Results at after intervention in the CT and control groups and effect-sizes.

	Experimental (*n* = 9) mean ± SE	Control (*n* = 8) mean ± SE	Between groups *P*	Within groups *P*	Interaction *P* ^1^	Effect-size (Cohen's *d*)
Positive and negative syndrome scale for schizophrenia						
Negative subscale						
Baseline	11 ± 4	11.7 ± 3	0.303	0.358	0.753	0.877
Post	11.4 ± 4.1	15.3 ± 7.5				
Positive subscale						
Baseline	12 ± 3.3	11.2 ± 3.1	0.610	**0.012**	0.935	0.000
Post	10 ± 3	9.2 ± 2.2				
General psychopathology subscale						
Baseline	24.4 ± 3.7	25.7 ± 4.1	0.576	**0.044**	0.581	0.486
Post	23 ± 3.3	22.7 ± 4.3				
Total score						
Baseline	47.4 ± 8.4	48.7 ± 7.8	0.599	0.275	0.760	0.263
Post	44.4 ± 6.7	47.3 ± 12.5				

Neuropsychological measures						
Trail Making A						
Baseline	57.2 ± 41.2	59 ± 34	0.903	**0.003**	**0.019**	0.821
Post	49.2 ± 26.5	40 ± 21				
Trail Making B						
Baseline	155.8 ± 89.3	121.1 ± 60	0.900	**0.045**	0.427	0.272
Post	141.5 ± 153.8	128.5 ± 98.9				
Stroop Test Card I						
Baseline	18.5 ± 4.6	19.2 ± 8.3	0.756	0.343	0.799	0.691
Post	19.1 ± 8.5	17 ± 4.0				
Stroop Test Card II						
Baseline	28.1 ± 15.4	22.2 ± 10.5	0.334	**0.001**	0.930	0.015
Post	23.6 ± 15.3	17.8 ± 5.7				
Stroop Test Card III						
Baseline	29.8 ± 8.9	27.6 ± 9.9	0.958	0.553	**0.036**	1.549
Post	27.5 ± 7.1	31.8 ± 10.3				
MWCST total categories						
Baseline	4.3 ± 1.9	4.1 ± 1.7	0.562	0.478	0.067	1.177
Post	3.4 ± 2.1	4.5 ± 2.5				
MWCST perseverative errors						
Baseline	8.1 ± 9.6	8 ± 9.9	0.549	**0.046**	0.395	0.134
Post	3 ± 2.9	1.8 ± 3.4				
MWCST failure to maintain set						
Baseline	0.3 ± 0.5	0.6 ± 0.7	0.702	0.745	**0.010**	1.086
Post	1.2 ± 1.3	0.3 ± 0.5				
Logical memory I						
Baseline	14.4 ± 8.3	13.7 ± 10.8	0.854	0.468	0.680	0.199
Post	14.7 ± 7.3	14.7 ± 10.7				
Logical memory II						
Baseline	10.6 ± 6.6	8.8 ± 11	0.802	0.643	**0.003**	1.618
Post	7.3 ± 8.1	13 ± 10.7				
Visual reproduction I						
Baseline	34 ± 8.9	29.7 ± 9.7	0.378	0.157	0.404	0.261
Post	29.8 ± 11.7	27.1 ± 11.5				
Visual reproduction II						
Baseline	28.6 ± 9.9	13.5 ± 9.1	0.053	**0.072**	0.103	1.127
Post	29.1 ± 12	23.5 ± 15.1				

^1^Interaction: groups × time.

SE: standard error.
